# Strengthening the capacity of managers in pharmaceutical services based on Primary Health Care (PHC) at different levels of the health system

**DOI:** 10.1186/1478-4491-12-34

**Published:** 2014-06-13

**Authors:** Isabel Cristina Martins Emmerick, Luisa Arueira Chaves, Nelly Marin, Vera Lucia Luiza

**Affiliations:** 1 Department of Population Medicine, Harvard Medical School, Harvard University (DPM/HU), Boston, MA, USA; 2 Center for Pharmaceutical Policies, Sergio Arouca National School of Public Health, Oswaldo Cruz Foundation, 1480, Rua Leopoldo Bulhões, #624, Manguinhos, Rio de Janeiro 21041-210, Brazil

**Keywords:** Distance learning, Primary Health Care, Pharmaceutical services, American region, Continuing professional development

## Abstract

**Introduction:**

Distance learning methods have been widely used because of their advantages to continuing professional development processes. The Primary Health Care (PHC) is a strategy which has been implemented in order to improve the efficiency of health systems. Due to the need for access to medicines and technologies regardless of the strengthening of health systems, a new approach that better integrates both pharmaceutical services and health systems has been implemented.

**Case description:**

This is a case study which consists of describing the process of restructuring, developing and implementing the second version of the Virtual Course on Primary Health Care-based Pharmaceutical Services for managers (CVSERVFAPS-12). The main objective is to strengthen the capacity of managers in pharmaceutical services, based on PHC at different levels of the health system, in order to support the restructuring and empowering of these services and, consequently, the health systems in the American region.

**Discussion and evaluation:**

Many evaluation tools were applied to identify the achievement/improvement of planned competencies. The intervention proposals were collectively built and intended to be more than an academic exercise, looking forward to being implemented as a strategic intervention to promote changes in the pharmaceutical services of the American region. The main strengths identified for the second version of the course were related to the quality of the didactic material and content. Additionally, the tutors’ support was commented upon as a positive aspect. The main challenges faced in this rebuilding process related to the due dates of the activities and lectures as well as the time to capture and assimilate the content.

**Conclusions:**

The CVSERVFAPS-Pilot was reformulated and CVSERVFAPS-12 is consistent with the issues raised in the pilot course’s evaluation in 2011, which were successfully implemented. The use of the distance learning strategy, through a virtual environment, for the application of the Virtual Course on PHC- based pharmaceutical services for managers, is appropriate and confirmed its role in public policy promotion through effective retention and distribution of health workers.

## Background

The distance learning method is recognized as a way to facilitate access and put into effect the process of continuing professional development (CPD) as well as to adapt to political changes, current situations and technological advances. Besides, it reaches the entire target population and encourages the CPD process as the training develops without taking the learners out of their work environment. This makes it easier for them to connect the concepts learned with their daily practice [1].

More recently, the learning process in the virtual environment, as part of the learning network, has been rapidly growing [2]. In this type of approach, learners acquire new forms of interaction that facilitate active learning, which supports the development of individual and group skills and increases their ability to build a collective knowledge and learn from each other’s experiences in a participative way [3].

An important advantage of distance learning is its viability for the health field, mainly in low-resource settings. The learners can continue to work while upgrading themselves, ensuring that their health services will not experience staffing challenges as a result of the health care workers’ participation in the training [4].

The Primary Health Care (PHC) is a strategy that has been implemented in order to improve the efficiency of health systems. However, many things have changed since Alma-Ata. The PHC concept proposed in 1976 expressed a broader sense of PHC, making it responsible for coordinating the health system and having as a crucial concept the Social Determinants of Health [5].

However, over time, PHC has been implemented very differently between countries, especially in the Latin American region. In some cases, the PHC concept has been distorted, becoming a vision of ‘selective’ care, targeting specific sub-populations, or as vertical disease-specific programme [5].

According to that critical view, in 2007, the Pan American Health Organization (PAHO) published the position paper entitled ‘Renewing the Primary Health Care in Americas’, reaffirming the broader Alma-Ata concept and stressing the PHC as the basis and fundamental strategy to develop a national health system aiming to achieve the fundamental human right to health. As part of this movement, a new approach has been sought in order to better integrate pharmaceutical services (PS) and health systems, since access to medicines and technologies is one of the necessary elements to strengthen health systems [6].

In 2009, the Primary Health Care-based Pharmaceutical Services Working Group (PHCbPSWG) was created in the American region, composed of experts in pharmaceutical policies and/or PHC from different countries aiming to support the restructuring and strengthening of the pharmaceutical services based in PHC. The composition of this working group tried to ally managerial, health service and academic expertise. One of the strategies adopted by this group was the development and implementation of a position paper establishing the basis of the proposal advocated by PAHO. Another strategy was an online course to promote this new approach using PAHO’s platform, the Virtual Campus of Public Health (VCPH), supported on the Moodle platform [7].

The distance learning tool was chosen to reach as many leaders and managers of PS in the Latin American and the Caribbean regions as possible, in order to improve their capacity to develop an integrated PHC-based PS. In the strategic process of strengthening the pharmaceutical services in the Americas proposed in the course approach, the VCPH was chosen as the virtual environment because of its methodological proposal of collective construction among the countries involved.

Chosen through having developed the course, the group began by sketching up a pilot experience for its first version. During a meeting of the PHCbPSWG held in Porto Alegre on May 2010, the course proposal was outlined and a subgroup composed of professors was responsible for developing the pilot course. Under the general coordination of PAHO and the academic coordination of the Nucleus of Pharmaceutical Policies, the production of the pedagogic material of the course was developed. The group chosen for the task was composed of six professionals from PAHO offices, universities and research institutions, all of them involved with the theme of PHC and PS.

The course used a practice-based approach to teaching, consistent with the educational strategy of the VCPH. Three tutors were recruited and trained to apply the course. They had to have a college degree, teaching experience, desirable experience in distance learning and be from the field of pharmacy. The curriculum was organized in modules and learners were required to design an intervention proposal based on a problem selected from each participant’s workplace or practice site. The target audience was composed of professionals involved in management, administration and technical activities in PS, academics in public health and pharmacy education, PHC professionals in the Ministry of Health, professionals involved in pharmacy service delivery and those involved in pharmaceutical services delivery research.

Therefore, the pilot course of PS based on Renewed PHC (CVSERVFAPS-Pilot) was carried out between July and November 2010. In March 2011, it was held a review meeting for the pilot evaluation where strengths, weaknesses, and corrective interventions were identified and implemented in a following course in 2012.

Thus, this paper consists of a case study aiming to describe the experience of restructuring and implementing the second version of the Virtual Course on Primary Health Care-based Pharmaceutical Services for managers (CVSERVFAPS-12) for managers in the American region.

### Case description

An evaluative meeting occurred in Rio de Janeiro on March 2011, involving the general and academic coordination teams, professors, tutors and learners’ representatives. During a five-day interaction the pilot version was assessed, having as a support the compilation of all evaluation documents. Strengths, weaknesses and suggestions raised during the meeting are summarized in Table 1. In general, this evaluation pointed out that the CVSERVFAPS-Pilot objectives were successfully accomplished. However, some aspects needed improvement for the next edition, such as the reformulation of a theoretical teaching model, complete restructuring of the transversal module and the translation/adaptation of course material into Portuguese.

**Table 1:** Strengths, weaknesses and suggestions raised by the participants of the Primary Health Care-based pharmaceutical service pilot version course

**STRENGTHS**	**WEAKNESSES**	**SUGGESTIONS**
• The transversal module allowed having intervention proposals at the end of the course	• The transversal module presented many kinds of problems	• The intervention proposal transversal module must be kept but it should be modified to become better adjusted to the course needs and better connected with the other modules
• The teachers worked on topics of their expertise, which helped to achieve adequate and interesting materials	• Low availability of the professor responsible on the transversal module	• Improve the definition of activities, stating more clearly the objectives and how to develop them
• It achieved the goal of stimulating students by the balance between teaching model and the materials used	• The transversal module did not really integrate with the rest of the course	• Amplify the use of problem-based education techniques
• The strategy of cross-checking materials among the professors was really helpful	• Lack of feedback between professors and tutors	• The use of more adequate tools, focusing on a broader perspective
• The interaction between the coordination and tutors was considered optimal	• No standardized evaluation methods and issues with the criteria applied	• Improve the interaction between professors and tutors
• The academic activities were generally good and deepened the proposed topics	• Low feedback between tutors and students	• Improve the timing of activities and organize a feasible schedule for both tutors and students
• General and academic coordination support	• Low interconnection across the modules	• The course content must be prepared with reasonable antecedence to facilitate the revision
• Information sharing among teachers and tutors	• Lack of time from some tutors to dedicate to the course, lack of expertise in the specific field of PHC	• Avoid the last trimester of the year to apply the course (to avoid holidays and vacations)
• The interaction and shared experience among the mentors	• Activities timing and feedback	• Improve the course layout and organization
• General course guidelines and modules helped the course methodology	• Lack of interaction among the students	• Improve the tracking of activity completion
• The training materials were very up to date and interesting	• Timing of material preparation; the uploaded materials were not uploaded at the appropriate time	• Improve tutor training on the course-specific material and content as well as the web platform
• The module ‘Pharmaceutical Service and its components’ was the most exciting by its use of multiple teaching strategies	• The synchronous session held in the Blackboard live session tool was a difficult to manage because of the participants' different time zones	• Periodic meeting involving coordination and tutors to follow up the modules
• The interaction among the whole team was excellent	• Lack of homogeneity among module structures	• Improve the evaluation tools
		• Incentivize more participative behavior among the students
		• Improve follow-up of the students’ performance during the course
		• Reduce the volume of reading for the module and focus on content integration
		• Increase professors' participation on their specific module

Rio de Janeiro, 2011.

**Figure 1:**
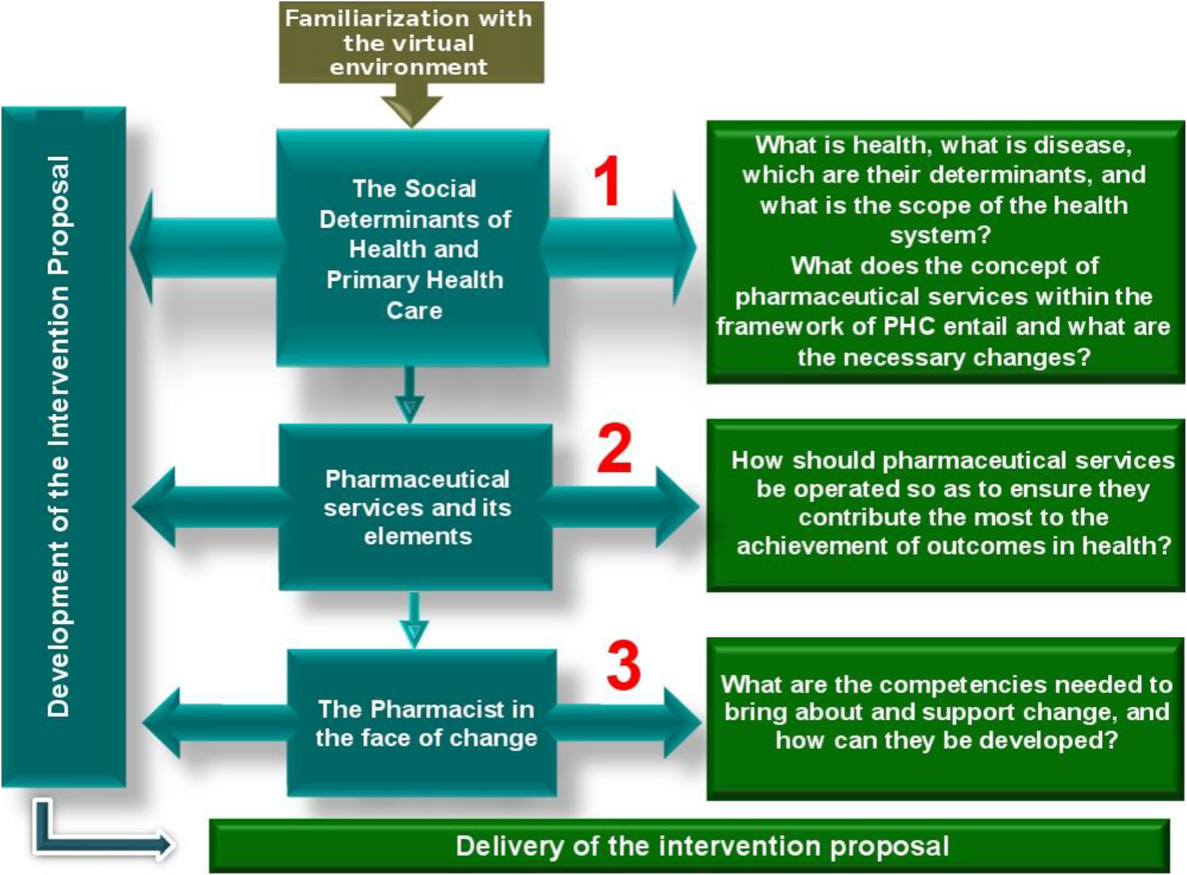
Virtual course on Primary Health Care-based pharmaceutical services for managers structure framework, 2012 version.

**Figure 2:**
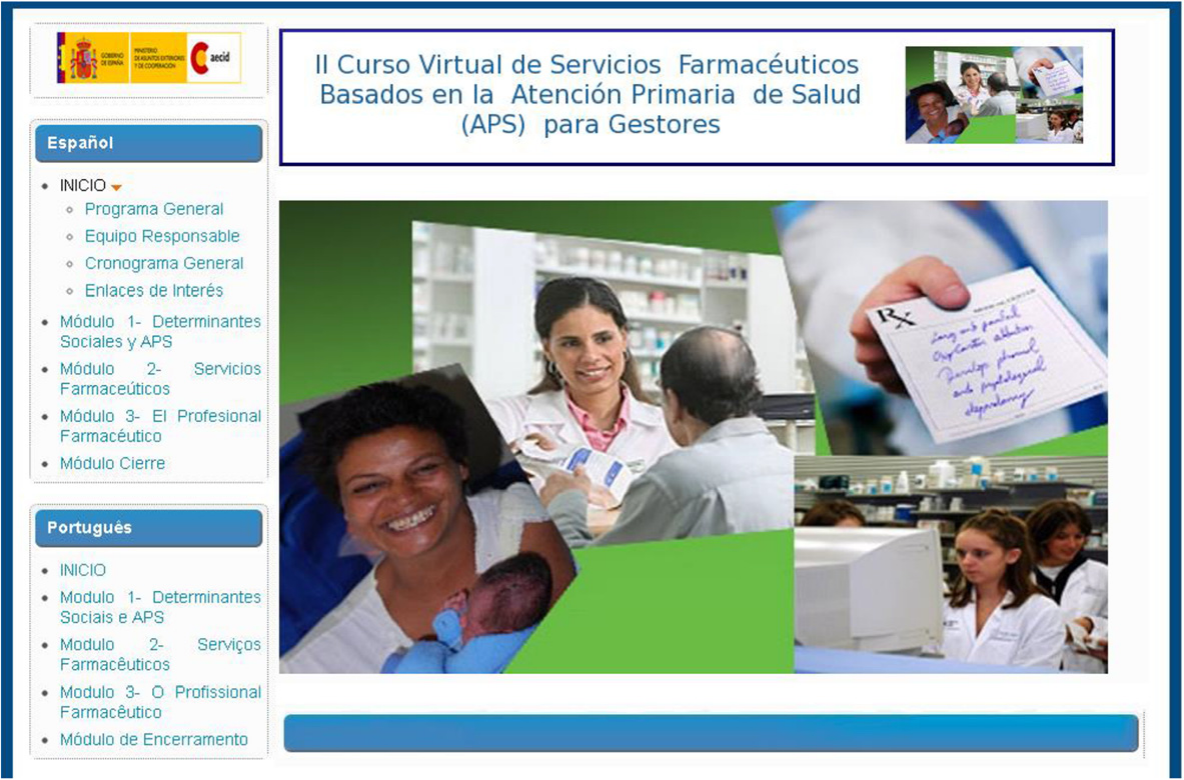
Virtual course on Primary Health Care-based pharmaceutical services for managers, 2012 version.

The course reformulation process involved two more professors which made a team of eight in the CVSERVFAPS-12. It was specially challenging because the professors were from four different countries and those from Brazil were from two different states. However, the virtual environment was very helpful in managing the distance and the work was developed under a collaborative and consensual perspective. All the materials produced were extensively reviewed and discussed. All the restructuring process involved several virtual meetings and lasted about ten months.The course was then reorganized in a three-module structure comprising sequential and transversal set of activities that lasted throughout the course. Under this transversal set of activities, the learners were invited to develop an intervention proposal for their country or local context (in Brazil’s case) (Figures 1 and 2). It was expected that the intervention proposals developed throughout the course could go beyond the academic exercise. It was intended that these proposals could be feasible enough to be implemented, resulting in changes in the practice of pharmaceutical services.

A broad set of learning strategies and activities was used. For example, playful activities including crosswords, puzzles and column-matching comprised part of Module 2 activities. Some interactive activities as a peer evaluation instrument, used in the proposal intervention construction and the 360° evaluation when discussing professional competencies also proved to be very useful in supporting the learning process.

Table 2 and Table 3 summarize the description of course’s didactic materials and academic administrative documents for both pilot version (2010) and second version (2012). These materials served as guides to the implementation and management of the course.

**Table 2:** Description of virtual course on Primary Health Care-based pharmaceutical services for managers' didactic materials pilot version (2010) and second version (2012)

**Name of the material**	**Objective**	**Content**	
		**Pilot version**	**Second version**
Programme	To present the course's purpose and development	• Welcome	• Welcome
		• Teaching and coordination team presentations	• Introduction
		• Duration and time commitment	• Teaching and coordination team presentations
		• General information	• Duration and time commitment
		• Purpose	• General information
		• Specific objectives	• Purpose
		• Competencies	• Specific objectives
		• Target audience	• Competencies
		• Requirements	• Target audience
		• Teaching strategies	• Requirements
		• Evaluation of students, tutors and modules	• Teaching strategies
			• Operational aspects
			• General course schedule
			• About synchronous interactions
			• Evaluation of students, tutors and modules
Module's study guide	To present the contents and activities to be developed in the module	• Objectives: general and specifics	• Introduction
		• Teaching strategies	• Objectives: general and specifics
		• Readings: required and further	• Competencies
		• Description of activities	• Teaching strategies
			• Readings: required and further
			• Content development
			• Module assessment
			• Description of activities
			• Timetable
			• Tools
Module's correction guide	To standardize the activities' correction criteria among tutors	• General consideration	• General consideration
		• Objectives	• Objectives
		• Evaluation of required activities	• Evaluation of required and supplementary activities
		• Feedback to students	• Criteria for evaluation the tutorial sessions
		• Feedback schedule	• Feedback to students
Module's presentations	To present its main concepts and activities	• Inexistent	• According to each module

**Table 3:** Description of administrative documents virtual course on Primary Health Care-based pharmaceutical services for managers pilot version (2010) and second version (2012)

**Name of the material**	**Objective**	**Content**	
		**Pilot version**	**Second version**
**Administrative materials**			
Terms of reference for professors	To describe the professors activities and responsibilities	• Available	• The pilot version was reformulated by including responsibilities during the course application
Terms of reference for tutors	To describe the tutors’ activities and responsibilities	• Available	• The pilot version was revised but major changes were made
Organizational aspects	To describe the academic management procedures	• Inexistent	• Introduction
			• Overview of course organization
			• General course schedule
			• Synchronous interaction schedule
			• Course forum
			• Knowledge resources (educational strategies)
			• Evaluation of participants
			• Operational procedures
Naming files	To guide on how to name activities' files	• Inexistent	• Naming activity product files
Managing tasks		• Inexistent	• Open Project

The weight of each module in the evaluation of student performance was based on the analysis of the relative importance of each topic regarding the overall objective of the course. It was decided by the professors who elaborated the modules. Each module has an evaluation guide called Correction Guide for the tutors to analyze the learners’ performance. All tutors and coordinators took the tutoring course offered by VCPH. In addition to the prior teaching experience of the tutors, they were trained with specific course material with the aim of testing, predicting and clarifying any questions that could arise during the implementation of the course.

A comprehensive evaluation process was applied during the course, comprising formative (in progress) and summative (final) approaches [8]. Learners were evaluated according to their competencies achievements through a set of activities targeting actual and contextualized problems and they were asked to self-evaluate. Also, tutors and learners were asked to fill a questionnaire evaluating the course. It is important to highlight that all these materials are under open access and all the teachers authorized their distribution and utilization after each version of the course was finished. The materials can be downloaded in the VCPH [9].

For CVSERVFAPS-12, which received financial support from the Brazilian Ministry of Health, 90 spots were offered and 6 tutors were hired, in order to obtain a ratio of 15 learners per tutor. All tutors, including the experienced ones, were trained together on the specific course content in a one-week workshop held in Medellin, Colombia in February 2012, with the professors’ participation. During this workshop all the academic materials were fully reviewed.

In advance, the tutors had received the Study Guide, the Correction Guide, the Operational Aspects Document, all the required and further reading and videos used as didactic complement. By studying this material in advance, they had the opportunity to raise doubts and clarify them during this period. Throughout this week, the contents were presented, various exercises were undertaken and comments and suggestions for the improvement of the material were collected.

Several management tools, besides the ones already utilized in the restructuring process, were implemented by the academic coordination, such as the use of OpenProject software to help managing and recording all the activities developed throughout the course and the implementation of a tutor performance feedback per module.

The routine activities were better systematized in relation to the pilot version and comprised: fortnightly meetings with the tutors and general coordination; follow-up of absent learners; evaluation of tutors’ performance; managing of opening and closing period of activities, module and evaluation; management of problems with the virtual environment and of the synchronous activities, as well as dealing with any unexpected issues that emerged throughout the course.

Another important task was to adapt and post all materials and activities onto the Moodle platform which involved splitting the study guides into different documents (for example, schedule, teaching strategies and tools, description of activities) in order to supply them to the student in a friendly and intuitive way. The programme of the Moodle platform included a set of tasks involving creating image files, processing editable files into a non-editable format (PDF or HTML), building of reference and counter-reference links for internal and external files, learning activities inclusion and programming (for example, posting deadlines, space for loading documents, adaptation of complete references to files leaving only the required pages for the learners’ reading). The course’s interface was opened one month in advance giving the chance for tutors and professors to familiarize themselves with its navigation and to test all the links and files. This gave the academic coordination the opportunity for early problem detection and problem fixing before the beginning of the course.

The target audience was kept the same as for the previous version. The importance of including at least two learners per country in order to promote a minimum critical mass of human resources qualified in this area was discussed since this new approach might prove quite innovative for some of them. All the required material, including the web site, was translated/adapted to Portuguese (the pilot version was only in Spanish). The number of seats was increased from 42 to 90, of which 30 were for Portuguese-speaking countries and 60 for Spanish-speaking countries of the region.

Finally, the second edition took place between May and August 2012. The course was coordinated by Nelly Marin, Regional Advisor on Pharmaceutical Policies of PAHO, who created and motivated the PHCbPSWG through the process, and had as academic coordinator Vera Lucia Luiza from the WHO-PAHO Collaborating Centre for Pharmaceutical Policies, also part of the National School of Public Health. All the professors were invited to participate along the course, especially to support the tutors in subjects related to module’s content and the correction of learners’ activities.

The total investment per learner, which includes the amounts spent for development/adjustments and application, was US$ 1,061.19 for the 2010 pilot course and US$ 388.46 for the 2012 edition. Considering only the application cost per learner, the amount spent was similar, being equal to US$ 439.7 and US$ 319.43 for 2010 and 2012, respectively.

The virtual course is a strategy to improve the PS in PHC that has increased in relevance and extent in the American region, being possible to be verified by the number of applications, learners selected, countries reached and intervention proposal projects throughout the different editions. For instance, the pilot course (2010) received 62 applications from learners from 16 countries and after the selection process, 42 learners were accepted and 34 concluded the course (80.9%). Learners were divided into three tutor groups. In contrast, the second version (2012) received 340 applications from learners from 19 countries and after the selection process, the 90 initial vacancies were expanded, and 98 learners were accepted. They were separated into six groups, being four in the Spanish language (66 learners) and two in Portuguese (32 learners). The drop-out rate on the CVSERVFAPS-12 was around 15% and 82 learners completed the course successfully. As in the pilot version, learners presented as final course activity an intervention proposal project (Table 4). Thus, in the second edition, 21 intervention proposals were developed. Five of them were made by Brazilian groups and 16 by Spanish-speaking countries, as follows: Argentina, Bolivia, Chile, Colombia, Costa Rica, El Salvador, Ecuador, Guatemala, Honduras, Mexico, Nicaragua, Panama, Paraguay, Peru, Dominican Republic, Uruguay and Venezuela.

**Table 4:** Intervention proposals titles per virtual course on Primary Health Care-based pharmaceutical services for managers version

**Country**	**Intervention proposal title**	
	**2010 version**	**2012 version**
Argentina	No participant this year	Pharmaceutical services organization of a public hospital reference area, which works in a network and includes private pharmacy pharmacists
Bolivia	Intervention strategy for the development of pharmaceutical care in the context of the Family and Intercultural Community Health based national health system	Implementation strategy of pharmaceutical care-based municipal pharmacies that comprise the Family and Intercultural Community Health based national health system model
Brazil	Pharmaceutical services structuring through the implementation of the model ‘Farmácia da Bahia’ in Bahia municipalities with up to 15,000 inhabitants	Educational activities for the older aged and healthy aging: an intervention in a PHC unit
		Health community agent promoters of medicines' rational use
		Disseminating renewed PHC-based pharmaceutical services vision to professional pharmacists
		Use of the pharmaceutical services management national system (HORUS) for pharmaceutical services qualification in Brazil’s municipalities
		Centralized drug purchase by the Brazilian Ministry of Health
Chile	No participant this year	Increasing elderly adherence therapy in Valdivia city
Colombia	A proposal to make the pharmaceutical services an integral part of health services in the Bogotá health care model development	Designing a pharmaceutical service provision model based on renewed PHC, focusing on integrated health care networks in Cartagena de Indias local hospital
Costa Rica	Promoting correct and rational use of medicines through the implementation of PHC-based pharmaceutical services	Strategies to implement an integrated pharmaceutical care network for patients with hypertension and/or diabetes in the pharmaceutical services of the Costa Rican National Insurance Institute
Dominican Republic	No participant this year	Transforming pharmacists’ professional culture towards being PHC oriented
Ecuador	Promoting rational use of medicines in the community	Comprehensive care of patients with chronic metabolic diseases
El Salvador	Contribution to improve the quality of pharmaceutical services provided by the Ministry of Health and Social Assistance through the implementation of PHC focused dispensing	Strengthen ‘La Presita San Miguel’ colony and Atlacatl household delivery programme based on the objectives of renewed PHC
Guatemala	No participant this year	Implementation of pharmaceutical care service in a specialized palliative care department of the cancer institute ‘Dr. Bernardo valley’-Incan-Guatemala
Honduras	Pharmaceutical service reorientation to the individual, family and community of the Honduran institute of social security center for comprehensive care of the elderly	PHC in peripheral pharmacy services in International Health Services clinic # 3
Mexico	Training course for those responsible for the bidding process for drugs in the Mexican states	Access and availability of opioid analgesics in Mexico: design and implementation of strategies to increase the quality of life of patients with difficult-to-control oncological and non-oncological acute or chronic severe pain
Nicaragua	No participant this year	Pharmaceutical care in pharmaceutical services of Leon municipality health units
Panama	No participant this year	Integration of the pharmacist in renewed PHC
Paraguay	No participant this year	Renewed PHC-based pharmaceutical services for hypertension monitoring and detection
Peru	Improvement of outpatients’ economic, clinical and human outcomes through pharmacotherapy follow-up	Pharmaceutical intervention based on renewed PHC in elderly patients, in Ministry of Health specialized health centers
Uruguay	No participant this year	Health promotion and prevention development programmes in the control of hypertension
Venezuela	Pharmaceutical services based on renewed PHC improvement and professional training programme	Venezuela's pharmaceutical services healthy life styles promotion programme design

### Discussion and evaluation

Due to the collaborative process of restructuring the course, the experience was enriching because of the active involvement of stakeholders from different institutions and countries with their own realities, knowledge and expectations. We consider that this was really a continuous professional development experience [10,11] since we used a competence improvement approach throughout the course, stressing the competencies required to enable the learners to adapt themselves and their context to the changes from a traditional PHC to the renewed model. The inclusive and participatory discussion performed by the general coordination (PAHO) was an important factor to the group’s cohesion and success achieved so far. The support of Brazil’s Ministry of Health was vital for the realization of CVSERVFAPS-12.

**Figure 3:**
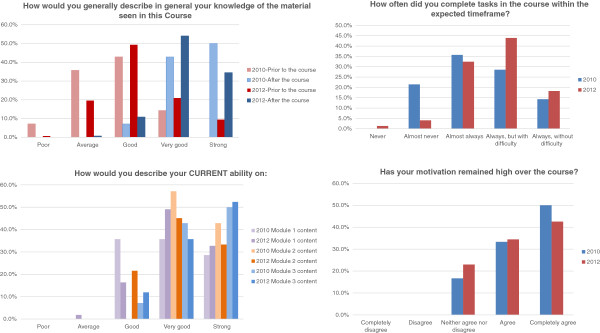
Virtual course on Primary Health Care-based pharmaceutical services for managers pilot version (2010) and second version (2012) evaluation results.

The evaluation process of the course by the students changed from the CVSERVFAPS 2010 Pilot Course to the final version CVSERVFAPS 2012. The first one had three evaluative moments: the beginning, the halfway and a final evaluation at the end of the course; this first version included questions relating to each module. The first assessment comprised learners’ profile, main work activity, expectations and challenges that they expect to fac during the course. In the second version - CVSERVFAPS-12 - an evaluation step was introduced during the Familiarization Module with regards to the learners’ knowledge about the virtual environment and tools. The first assessment was kept, and the module evaluations were performed at the end of each one, allowing ongoing adjustments.All assessment tools were composed by closed-ended questions, using a five-points scale, but with a final space to allow open comments. The average of learners who answered the evaluation forms in 2010 was 33.3% (14 learners of 42) and in 2012 around 58% (50 learners of 86) gave feedback about the course. In both course editions they pointed out (Figure 3) an increase in their knowledge in all topics, and the majority of them felt that they ended with increased (very good or strong) ability in the course contents. The learners found the dynamic appropriate and remained motivated during the course, mostly in the 2012 version. In 2010, a common complaint was about the total number of activities and due dates for the tasks, addressed by the question: ‘How often did you complete tasks in the course within the expected time-frame?’ with ‘Almost never’ being the most frequent answer in 2010. In 2012, after the reformulations had been implemented, the most frequent answer was: ‘Always, but with difficulty’.

Despite the improvements, some problems remained and the main challenges faced by the CVSERVFAPS-12 concerned the due dates of the activities and lectures as well as the time to capture and assimilate the content. Other aspects mentioned were the difficulties in attending the synchronous meetings and the initial adaptation process on the course methodology. The difficulties regarding the synchronous meeting were mainly related to: managing office time with the time scheduled for it and using the tool of the synchronous meeting software used in VCPH, the Blackboard room® (http://www.blackboard.com).

The tutors evaluated this experience as successful and mentioned that the activities allowed the learners to correlate their work activities with the theory in a very practical manner. Positive features mentioned included the support of the academic coordination through the forums and regular meetings, the mandatory and further readings, the videos and the playful activities of Module 2 and the importance of the asynchronous forums for discussion and exchange between learners from different countries. In addition, the tutors highlighted the importance of the synchronous discussion per tutor group (either in the mandatory tutorial session or in the tutor office hours in which the learners participation were not mandatory) in contrast with the entire group meeting. It was said that the first ones had better participation and improved the bond between tutors and learners. However, it was mentioned that the tutor office hours’ goal was not clear to all of the learners.

The tutors also indicated where the course could be improved. The main points included the addition of readings in order to better support the learners on some issues, reorganization of module’s duration according to the number of activities, improvement of evaluation criterion of some activities, better organization of forum topics, more time or orientation for learners on the use of virtual tools (specially the synchronous sessions tools) and, finally, the revision of correction guides, emphasizing those aspects regarding the asynchronous forums.

The coordination evaluated the course as good in accomplishing its objective but realized that there was still room for improvement, especially regarding the duration of modules, tutors’ performance feedback time span and correction mechanisms of Module 2 external activities. As a result of this evaluation it was decided to remove one activity from Module 3, increase by two weeks the wrap-up module duration, deliver tutors’ performance feedback every two weeks instead of every module, and, lastly, the addition of new steps or integration of Module 2 external activities into the Moodle system.

The comments and suggestions sent from the learners were considered in the course general evaluation and were reviewed during the elaboration of the 2013 version.

The intervention proposals are the main product and aims to strategic planning an intervention for promoting change of pharmaceutical services and were a strength in both pilot and 2012 versions, it being remarkable that most of them target the individual, family and communities.

Applying the CVSERVFAPS simultaneously in two languages, despite being a challenge to the course management and execution, is worthwhile. The course was successfully adapted to the English language and the 2013 version will include most languages in the American continent.

However, the applicability of this approach in other contexts outside the Americas is yet to be tested. From our experience, when applying the course in other scenarios it is important to critically appraise the didactic material (available at the VCPH) in order to adapt any examples or tools to social and cultural aspects of the context in where is intended to be applied. Today, the material (didactic and bibliography) is available in Portuguese, Spanish and in English, and, therefore, for application in countries with other languages it would be necessary to translate/substitute it and adapt some bibliographies.

Since this course was developed for the virtual environment, the limitations of this approach include struggles with poor access to good quality (or any at all) Internet connection. This is particularly important when dealing with low resources settings. Still, regarding technological challenges and considering the fast development of virtual and communications tools, this course is a ‘hostage’ of its historical period. This means that with the development of new virtual tools and knowledge evolution, the didactic material and strategies must be updated.

Another limitation concerns the target population and theme addressed in this course. That specificity makes it difficult to predict if the strategies chosen can be applied for other health professionals or even to work other concepts. This dimension is important as the professionals who developed the material are mostly from the Latin American region and therefore share at some level, cultural, historical and symbolic aspects. Therefore, their teaching approaches may not be the best for other cultural environments, stressing the importance of cultural appraisal and adaptation of the materials to other experiences.

Finally, these limitations and comments on generalization, express the process described in this paper. The development and application of the CVSERVAPS-12 was only possible due to the collective commitment of the professorial team, tutors, academic and general coordination, and their willingness to be perpetual learners, by revising, discussing, listening and making the required changes and corrections together during every step of its development.

When comparing the CVSERVAPS-12 experience with the scientific literature, several papers [2,3,12-16] report and describe cases of implementing distance learning strategies as a way of improving health service quality and, consequently, the health systems. In the literature there is a consensus that the distance learning process yields good results towards achieving its objective [3,17,18], which reinforces the use of the virtual environment as well as Internet communication tools as a quicker and cheaper way of upgrading health professionals’ knowledge and ability to think in new alternative ways to solve everyday problems in health services and systems.

We have not found any papers describing or reporting a distance learning course on the PS theme; neither have we found any reports on any virtual course on this theme with that degree of coverage in terms of countries and diversity of learner profile. Thus, within that context, this course is unique and has a strong potential in changing the pharmaceutical services paradigm towards an individual, family and community centered PS in the American region [6].

The use of educational technologies is undoubtedly a societal demand and need in order to qualify systems and health services. The advancement of information and communication technologies is resonating with modes of human action, since knowledge is required to build up the relationships between science, education, society and work. The search for alternatives to offer education to articulate the context and broad scope challenges the trial methodological possibilities and organizational processes of teaching and learning that strengthen the individual and collective initiatives which focus on solutions to health systems everyday problems.

## Conclusions

The supporting tools and materials developed were useful in really helping the coordination work. The course had a low rate of absenteeism and most learners concluded with a distinction grade. All learners participated in the development of an intervention proposal.

The second course is consistent with the issues raised in the pilot course’s evaluation in 2010. The results achieved so far are in concordance with its proposal. The use of the distance learning strategy, through a virtual environment for the application of the Virtual Course on PHC-based Pharmaceutical Service for managers, is appropriate and confirmed its valuable role in public policy strengthening.

## Abbreviations

CVSERVFAPS: Virtual Course on Primary Health Care-based Pharmaceutical Services based for managers; PAHO: Pan American Health Organization; PHC: Primary Health Care; PHCbPSWG: Primary Health Care-based Pharmaceutical Services Working Group; PS: pharmaceutical services; VCPH: Virtual Campus of Public Health; WHO: World Health Organization.

## Competing interests

The authors declare that they have no competing interests.

## Authors’ contribution

ICME and LAC have made substantial contributions to the conception and design, or acquisition of data, or analysis and interpretation of data; have been involved in drafting the manuscript or revising it critically for important intellectual content; NM and VLL have made substantial contributions to the conception, design and critical revision for important intellectual content of the paper; (Group CVSERAPS-2012) - course development and professorial team; have made substantial contributions to the conception and design of the paper and all authors revised and approved the final manuscript. All authors participated sufficiently in the work to take public responsibility for appropriate portions of the content.

## Authors’ information

NM is an independent consultant, was the general coordinator in the pilot course and in the CVSERFAPS-12; VLL is a researcher at the Center for Pharmaceutical Policies, Sergio Arouca National School of Public Health, Oswaldo Cruz Foundation, Rio de Janeiro, Brazil (NAF/ENSP/Fiocruz) and was the academic coordinator of the course; ICME is a fellow researcher in pharmaceutical policies at Department of Population Medicine Harvard Medical School Harvard University (DPM/HU) and was part of the academic coordination in the 2012 version; LAC is a visiting researcher at the Center for Pharmaceutical Policies, Sergio Arouca National School of Public Health, Oswaldo Cruz Foundation, Rio de Janeiro, Brazil (NAF/ENSP/Fiocruz); CVSERAPS Group 2012 - Nelly Marín (PAHO); Vera Lucia Luiza (NAF/ENSP/FIOCRUZ); Isabel CM Emmerick (NAF/ENSP/FIOCRUZ); Adriana M Ivama Brummell (PAHO), Lesly Bustamante (COHAN), Mauro Castro (UFRGS); Rosa María Borrell (OPS/OMS); Víctor Ramírez (COHAN) were professors in the version 2012. Luiza Arueira Chaves was part of the academic coordination.

## References

[B1] Muller AK La educación a distancia como opción metodológica para el desarrollo de procesos permanentes de educación para los recursos humanos en salud; [Education at distance as a methodological option for the development of continuing education processes for human resources in health] Educ Méd Salud OPS 1987 21 56 69 2441950

[B2] Jardines Méndez JB Education in network: it means much more than distance education. Experience of Cuban medical colleges Educ Méd Super 2006 20

[B3] de la Valcárcel BCG Ledo MV Diego Olite FM The Cuban experience in health promotion diploma’s studies in the virtual public health campus Educ Méd Super 2013 27 12 24

[B4] Nartker AJ Stevens L Shumays A Kalowela M Kisimbo D Potter K Increasing health worker capacity through distance learning: a comprehensive review of programmes in Tanzania Hum Resour Health 2010 8 30 10.1186/1478-4491-8-30 21194417 PMC3023774

[B5] (PAHO/WHO) Pan American Health Organization/World Health Organization Why Renew Primary Health Care? Renewing Primary Health Care in the Americas: A Position Paper of the PAHO/WHO 2007 Washington, DC: Pan American Health Organization/World Health Organization

[B6] (PAHO/WHO) Pan American Health Organization/World Health Organization Definition, Mission, Vision, Values and Principles of PHC-based Pharmaceutical Services. Pharmaceutical Services Based on Primary Health Care: Position Paper Document of PAHO/WHO 2013 Washington DC: Pan American Health Organization/World Health Organization

[B7] Virtual Campus of Public Health (VCPH) http://www.campusvirtualsp.org/

[B8] Raupach T Brown J Anders S Hasenfuss G Harendza S Summative assessments are more powerful drivers of student learning than resource intensive teaching formats BMC Med 2013 11 61 10.1186/1741-7015-11-61 23497243 PMC3635879

[B9] Virtual Course on Primary Health Care-based Pharmaceutical Services for managers (CVSERVFAPS-12) http://cursos.campusvirtualsp.org/course/view.php?id=134

[B10] Martín-Zurro A Sala AG Desarrollo Profesional Continuo individual vs Formación Continuada Educ Méd 2005 8 http://dx.doi.org/10.4321/S1575-18132005000500001

[B11] Alentá HP Desarrollo profesional continuo, ¿de qué estamos hablando? Educ Méd 2008 11 53 56

[B12] Struchiner M Roschke MA Ricciardi RMV Ongoing, flexible distance learning through the Internet: course on decentralized management of human resources in health care Rev Panam Salud Pública 2002 11 158 165 11998181 10.1590/s1020-49892002000300004

[B13] Nunes TWN How can distance education contribute to comprehensive health practice? Rev Bras Educ Méd 2013 34

[B14] de Bicalho RNM de Oliveira MCL O processo dialógico de construção do conhecimento em fóruns de discussão Nterface - Comunic Saude Educ 2013 16 469 483

[B15] de Lima JVC Turini B Carvalho BG de Nunes EFPA de Lepre RL Mainardes P Cordoni Junior L Continuing education in health as a pedagogical strategy to transform the practice: possibilities and limits Trab Educ E Saúde 2010 8 207 227 10.1590/S1981-77462010000200003

[B16] MezzariI A IserII I WiebbellingII AMP TaroucoI L The Moodle as a reinforcement to classroom teaching of course parasitology and mycology in undergraduate medical course Rev Bras Eduação Méd 2012 36 557 563

[B17] Sherk KE Nauseda F Johnson S Liston D An experience of virtual leadership development for human resource managers Hum Resour Health 2009 7 1 10.1186/1478-4491-7-1 19133140 PMC2631468

[B18] Rangel-S ML de Oliveira BA Riccio NCR de Souza JS Redes de aprendizagem colaborativa: contribuição da Educação a Distância no processo de qualificação de gestores do Sistema Único de Saúde - SUS Comun Saúde Educ 2012 16 545 555

